# Death by Chloroform

**Published:** 1890-04

**Authors:** 


### DEATH BY CHLOROFORM.

The report of the Hyderabad Commission on the *modus operandi* of the lethal dose of chloroform, has given rise to much discussion. We have already given the essential fact apparently established by this Commission, viz., that in death from chloroform the first effect is upon the respiration, the heart ceasing to beat later.

We have received a letter from Dr. H. Culbertson, of Zanesville, O., in which he asserts that this view was clearly established by him in a series of experiments upon dogs and pigs, extending over four years, the results being published in 1861 ("Transactions of the Ohio State Medical Society," 1861, p. 35).

Dr. Culbertson's experiments were fourteen in number, and were apparently conducted with care.

Conclusion one is as follows: "That from the effect of chloroform by inhalation, the lungs will cease to act first, and the heart last."

Conclusion thirteen: "The fact that the lungs cease to act first and the heart last, sometimes one being more congested and again the other, would indicate that these organs are affected through the same channel, and not the heart solely at fault."

Conclusion fourteen is, "That restoration may take place after the heart ceases to beat, by the institution of artificial respiration."

Dr. Culbertson's views are thus seen to have been distinctly and categorically stated, and to have been based upon experimental evidence.

It is not true, however, that the opinion that chloroform kills by paralyzing the heart first, has been uniformly taught. In a number of standard works, it is admitted that respiratory failure often occurs first.

But it is now asserted that this must always be the case when chloroform is carefully administered, and that primary heart-failure, if it occurs, is due to shock or some accidental reflex influence.

We take pleasure in placing on record again the experiments and conclusions of Dr. Culbertson, for which he is entitled to great credit.—*Medical Record.*

